# Long-term effectiveness and safety of ustekinumab in Crohn’s disease: a prospective cohort study

**DOI:** 10.1097/MEG.0000000000002506

**Published:** 2022-12-22

**Authors:** Pepijn W.A. Thomas, Mark van Caem, Rachel L. West, Maurice G.V.M Russel, Jeroen M. Jansen, Tessa E.H. Römkens, Frank Hoentjen

**Affiliations:** Department of Gastroenterology and Hepatology; a Radboud University Medical Centre, Nijmegen; b Franciscus Gasthuis & Vlietland, Rotterdam; c Medical Spectrum Twente, Enschede; d Onze Lieve Vrouwe Gasthuis, Amsterdam; e Jeroen Bosch Hospital, ’s-Hertogenbosch, The Netherlands; f Division of Gastroenterology, Department of Medicine, University of Alberta, Edmonton, Canada

**Keywords:** Crohn’s disease, IBDREAM registry, real-world data, Ustekinumab

## Abstract

Real-world data showed that ustekinumab is an effective treatment for Crohn’s disease for up to 52 weeks. Yet, long-term effectiveness and safety outcomes beyond 52 weeks are limited. This study aimed to evaluate the corticosteroid-free clinical remission for up to 104 weeks. Secondary aims were focused on biochemical disease, dosing adjustments and safety outcomes.

**Methods:**

This multicentre prospective cohort study enrolled Crohn’s disease patients who started ustekinumab between May 2016 and September 2019. Participants had scheduled outpatient visits at week 0, 13, 26, 52 and 104. Data on clinical disease [Harvey Bradshaw Index (HBI) = 4 points = remission], biochemical disease (faecal calprotectin = 200 µg/g or C-reactive protein = 10 mg/l = remission), dose adjustments and adverse drug reactions (ADRs) were recorded.

**Results:**

We included 101 Crohn’s disease patients. In all patients, the proportion of patients in corticosteroid-free clinical remission was 35 and 36% at week 52 and 104. Of patients achieving corticosteroid-free remission at week 52, more than half maintained corticosteroid-free remission throughout week 104. Biochemical remission rates were 25 and 30% at week 52 and 104, respectively. In the first year of treatment, 33% required their first dose escalation, and 15% in the second year. Overall, 7% of patients discontinued ustekinumab due to ADRs. Ustekinumab persistency rates were 68% at week 52 and 59% at week 104.

**Conclusion:**

Ustekinumab is an effective and well-tolerated treatment for Crohn’s disease. More than half of all patients continued ustekinumab treatment after 104 weeks whereas one-third achieved corticosteroid-free remission.

## Introduction

Crohn’s disease is a disabling, chronic, immune-mediated inflammatory bowel disease (IBD) [1]. Biologic treatment of Crohn’s disease is primarily focused on inducing and maintaining remission [2]. The most recently approved biological for the treatment of Crohn’s disease, ustekinumab, is a fully human IgG1k antibody that blocks the p40 subunit of interleukin-12 and -23 [1].

The effectiveness and safety of ustekinumab for the treatment of Crohn’s disease patients were shown in the IM-UNITI trial [1]. In the 2-year extension of this trial, corticosteroid-free clinical remission rates at week 92 were 68% for ustekinumab at 12-weekly dosing and 63% at 8-weekly dosing, and this therapy was well-tolerated. Overall corticosteroid-free remission rate at week 92 was 59% when also including patients who required dose escalation [3]. Two main reasons limit the ability to extrapolate these findings to the real-world setting. First, this trial had strict in- and exclusion criteria. Consequently, most patients were not eligible for inclusion and therefore this study did not reflect IBD patients seen in daily practice [4]. Second, only patients who showed clinical response to induction therapy and benefitted from continued treatment at week 44, were able to participate in the 2-year extension trial [1]. These limitations warrant real-world data in which all Crohn’s disease patients starting ustekinumab can enrol and receive long-term monitoring.

Currently, several real-world cohort studies have assessed effectiveness and safety outcomes up to 52 weeks [5–16]. Corticosteroid-free remission rates after 52 weeks ranged from 12.2 to 47.4% [6,9,13,17,18]. Long-term outcomes beyond 52 weeks have only been assessed in a few real-world studies which were often restricted by small numbers or a retrospective design [19–22]. Recently, a large Dutch prospective cohort study reported a corticosteroid-free clinical remission rate of 34% at week 104 [17]. To provide more insight on the long-term real-world outcomes with ustekinumab in Crohn’s disease patients, we aimed to assess the proportion of ustekinumab-treated Crohn’s disease patients in corticosteroid-free clinical remission up to 104 weeks. Secondary aims were focussed on biochemical disease, dosing adjustments and safety outcomes.

## Methods

### Study design

This is a prospective multicentre cohort study using the IBDREAM registry. IBDREAM collects medical data from IBD patients’ electronic health records in four nonacademic and one academic hospital in the Netherlands as previously described [23–26]. Patients were monitored during regular outpatient visits which were scheduled at week 13, 26, 52 and 104 after treatment initiation. Patients were enrolled consecutively between March 2017 and September 2019.

### Participants

Patients ≥18 years were eligible for inclusion provided they had an established diagnosis of Crohn’s disease [27] and ustekinumab was initiated due to luminal or perianal disease activity. Patients received an initial weight-based intravenous dose according to the label (<55 kg: 260 mg; 55–85 kg: 390 mg; >85 kg: 520 mg) and subsequently an 8- or 12-weekly subcutaneous maintenance treatment with 90 mg at the discretion of the physician.

### Study variables

We collected baseline data regarding demographics, disease duration, disease location and behaviour consistent with the Montreal classification [28], previous and current IBD medication use, history of bowel-related surgery, Harvey Bradshaw Index (HBI), biochemical disease parameters including faecal calprotectin (FCP) and C-reactive protein (CRP) and radiologic and endoscopic assessment of disease activity, if available. During each outpatient visit, clinical disease activity was documented using HBI, and all changes in ustekinumab treatment and IBD medication were recorded. Furthermore, adverse drug reactions (ADRs) were recorded and labelled as probably, possibly, unlikely and not related. Infections were labelled mild, moderate or severe based on the need for treatment, as described previously [18]. Mild infections were self-limiting and did not require treatment. Infections were moderate if oral antibiotics or antiviral treatment were required and severe if hospitalization or intravenous administration of antibiotics or antiviral medication were required.

### Outcomes and definitions

The primary endpoint of this study was the proportion of ustekinumab-treated Crohn’s disease patients continuing ustekinumab at week 104 while in corticosteroid-free remission. Secondary endpoints included clinical remission, clinical response, biochemical remission, endoscopic disease activity, ustekinumab dosing adjustments, drug safety, discontinuation reasons and drug survival. Subgroup analyses for clinical and biochemical outcomes were performed for patients with clinically active disease at baseline and for patients previously exposed to vedolizumab, Clinical remission was defined as an HBI score ≤4 points. Corticosteroid-free remission was defined as clinical remission in the absence of systemic corticosteroids. Clinical response was defined as the reduction in HBI score of ≥3 points compared to baseline. Clinical response was only assessed in patients with clinically active disease at baseline (HBI ≥5 points). Biochemical remission was defined as a CRP ≤10 mg/L or FCP ≤200 µg/g. When data for the clinical disease was missing, patients were considered clinical nonresponders. The percentage of missing data for the HBI score per assessment ranged from 14 to 23%. When data for the biochemical disease was missing, patients were considered biochemical nonresponders. Endoscopic remission was defined as a Simple Endoscopy Score- Crohn’s disease ≤2 (absence of ulcerations). The safety endpoints involved the number and severity of ADRs and infections and overall hospitalizations per 100 patient-year. An ADR was defined as any unwanted or harmful reaction experienced by a patient following the administration of ustekinumab during the study, and possibly related to ustekinumab treatment [29]. Nonresponders were patients who discontinued ustekinumab treatment due to the lack of therapeutic effectiveness based on the physician’s decision. Secondary loss of response was defined as discontinuation of ustekinumab due to worsening of disease activity after initial response to ustekinumab within 3 months. Patients were censored in case of insufficient follow-up. Follow-up time was calculated from the date of the initial induction with ustekinumab until the cessation of ustekinumab or the end of follow-up.

### Statistical methods

Continuous variables descriptive statistics were reported as mean with SD for parametric variables, and median with interquartile range (IQR) for nonparametric variables. Categorical variables were expressed as number and percentage. Drug survival was described using the Kaplan–Meier curve. All ustekinumab discontinuation reasons were deemed as an event in the Kaplan–Meier curve, and patients were censored when lost to follow-up. IBM SPSS Statistics for Windows, version 26.0 (IBM Corp, Armonk, New York, USA) was used for the statistical analyses.

### Ethical consideration

The current study was reviewed and approved by the Radboudumc Committee on Research Involving Human Subjects (ref. 2018-4110). All enrolled patients signed informed consent for IBDREAM.

## Results

### Study population

In total, 101 Crohn’s disease patients were included and followed during 104 weeks of treatment. Two patients were lost to follow-up after 39 and 95 weeks, respectively. The median treatment duration was 104 weeks (IQR 41–104).

Baseline characteristics are shown in Table 1. Most patients were female (58%) and the median age was 40 years (IQR, 30–53). At baseline, patients had a median disease duration of 13 years (IQR 5–22) and 50% of patients had a history of intestinal surgery. Most patients had ileocolonic disease (61%), 49% had stricturing disease and 35% had penetrating disease. Nearly all patients were previously exposed to antitumour necrosis factor (TNF) agents (95%) and one-third to vedolizumab. Corticosteroids were used in 18% of patients with a median dose of 30 mg (IQR, 15–40). At inclusion, 67/101 patients (67%) had clinically active disease, 70/101 patients (69%) had biochemical disease and 46/49 (94%) had endoscopic disease. Similar baseline characteristics were observed for patients with clinically active disease at baseline.

**Table 1.** Baseline characteristics

		Total study population *N* = 101	Patients with clinical disease activity at baseline (*N* = 67)
Age, in years	Median (IQR)	39.9 (29.6–52.6)	37.8 (30.1–52.6)
Sex, female	*N* (%)	58 (57.4)	37 (55.2)
BMI, in kg/m^2^	Mean ± SD	25.4 ± 4.4	25.2 ± 4.6
Disease duration, in years	Median (IQR)	13.0 (5.2–22.4)	12.0 (4.5–24.0)
Disease location			
Ileum	*N* (%)	18 (17.8)	10 (14.9)
Colon	*N* (%)	20 (19.8)	15 (22.4)
Ileocolon	*N* (%)	62 (61.4)	41 (61.9)
Upper GI tract involvement	*N* (%)	12 (11.9)	9 (13.4)
Perianal disease	*N* (%)	26 (25.7)	17 (25.4)
Disease behaviour	*N* (%)		
Stricturing	*N* (%)	49 (48.5)	33 (49.3)
Penetrating	*N* (%)	35 (34.7)	22 (32.8)
Prior intestinal surgery	*N* (%)	50 (49.5)	34 (50.7)
Prior perianal surgery	*N* (%)	25 (24.8)	14 (20.9)
Prior anti-TNF			
≥1	*N* (%)	96 (95.0)	64 (95.5)
≥2	*N* (%)	61 (60.4)	37 (55.2)
Prior vedolizumab	*N* (%)	32 (31.7)	23 (34.3)
Prior anti-TNF and vedolizumab	*N* (%)	30 (29.7)	21 (31.3)
Disease activity			
Harvey Bradshaw Index score^a^	Median (IQR)	5 (2–9)	8 (6–12)
C-reactive protein, in mg/L^b^	Median (IQR)	11 (2–28)	8 (2–25)
Faecal calprotectin, in µg/g^c^	Median (IQR)	445 (190–1417)	516 (188–1598)
Endoscopic or radiologic-confirmed disease	*N* (%)	57 (56.4)	40 (59.7)
Concomitant medication			
Corticosteroids	*N* (%)	18 (17.8)	15 (22.4)
Corticosteroid dose	Median (IQR)	30 (15–40)	30 (20–40)
Immunomodulators	*N* (%)	31 (30.7)	22 (32.8)
Smoking status			
Active	*N* (%)	27 (26.7)	16 (23.9)
Previous	*N* (%)	22 (21.8)	14 (20.9)
Never	*N* (%)	48 (47.5)	34 (50.7)
Missing	*N* (%)	4 (4.0)	3 (4.5)

GI, gastrointestinal; IQR, interquartile range; TNF, tumour necrosis factor.

a Harvey Bradshaw Index score was documented in 81 patients (80%).

b C-reactive protein was measured at baseline in 88 patients (87%).

c Faecal calprotectin was measured at baseline in 65 patients (64%).

### Effectiveness outcomes

Effectiveness outcomes for patients previously exposed to vedolizumab are shown in Supplementary Files, Supplemental digital content 1, http://links.lww.com/EJGH/A821.

#### Steroid-free clinical remission

**Fig. 1.**
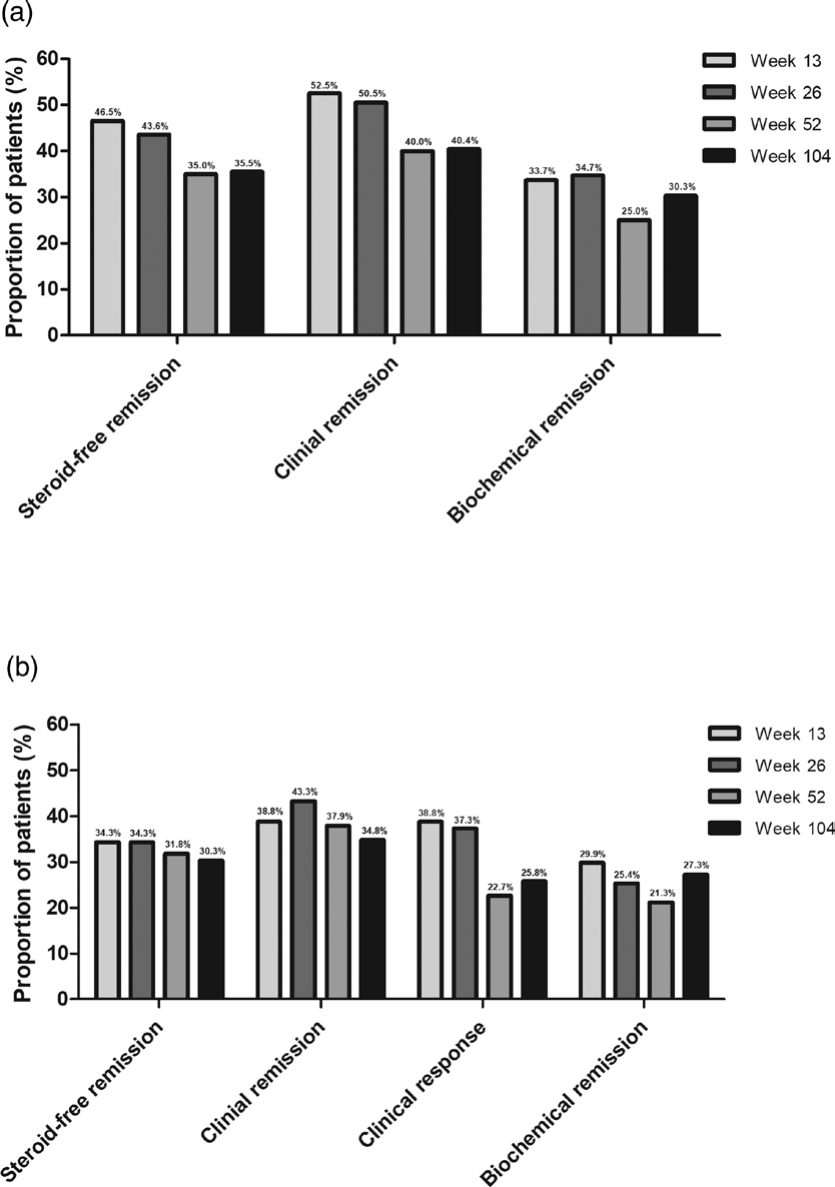
Clinical outcomes in (a) all ustekinumab-treated patients and (b) patients with clinically active disease at baseline defined as Harvey Bradshaw Index score ≥5. Proportion of patients with steroid-free remission, clinical remission, clinical response and biochemical remission at weeks 13, 26, 52 and 104. Clinical remission was defined as a Harvey Bradshaw Index score ≤4. Clinical response was defined as a least 3 points reduction in the Harvey Bradshaw Index score compared to baseline. Harvey Bradshaw Index scores were missing in 14–21% per assessment for the total patient group and 15–21% for patients with clinical disease activity at baseline.

Overall, the proportion of patients in corticosteroid-free clinical remission was 46.5% (*n* = 47/101), 43.6% (*n* = 44/101), 35.0% (*n* = 35/100) and 35.5% (*n* = 35/99) at week 13, 26, 52 and 104, respectively (Fig. 1a). Of the patients with corticosteroid-free clinical remission at week 52, 54.3% (*n* = 19/35) remained in corticosteroid-free clinical remission at week 104. In patients with biochemical disease activity at baseline (*n* = 70), corticosteroid-free clinical remission rates were 47.1% (*n* = 33/70), 44.3% (*n* = 31/70), 39.1% (*n* = 27/69) and 40.6% (*n* = 28/69) at week 13, 26, 52 and 104, respectively.

In patients with clinically active disease at baseline (HBI ≥5), the proportion of patients in corticosteroid-free clinical remission was, 34.3% (*n* = 23/67), 34.3% (*n* = 23/67), 31.8% (*n* = 21/66) and 30.3% (*n* = 20/67) at week 13, 26, 52 and 104, respectively. (Fig. 1b) Of the patients with corticosteroid-free clinical remission at week 52, 52.4% (*n* = 11/21) remained in corticosteroid-free clinical remission at week 104. In patients with biochemical disease activity and clinically active disease at baseline (*n* = 45), corticosteroid-free clinical remission rates were 37.8% (*n* = 17/45), 33.3% (*n* = 15/45), 34.1% (*n* = 15/44) and 36.4% (*n* = 16/44) at week 13, 26, 52 and 104, respectively.

#### Clinical remission and response

The proportion of patients in clinical remission was 52.5% (*n* = 53/101), 50.5% (*n* = 51/101), 40.0% (*n* = 40/100) and 40.4% (*n* = 40/991) at week 13, 26, 52 and 104, respectively. (Fig. 1a)

In patients with clinical disease at baseline (HBI ≥5), the proportion of patients in clinical remission was 38.8% (*n* = 26/67), 43.3% (*n* = 29/67), 37.9% (*n* = 25/66) and 34.8% (*n* = 23/66) at week 13, 26, 52 and 104, respectively. (Fig. 1b) Clinical response in these patients was achieved in 38.8% (*n* = 26/67), 37.3% (*n* = 25/67), 22.7% (*n* = 15/66) and 25.8% (*n* = 17/66) at week 13, 26, 52 and 104, respectively. (Fig. 1b)

#### Biochemical disease activity

The proportion of patients in biochemical remission was 30.7% (*n* = 31/101), 33.7% (*n* = 34/101), 34.7% (*n* = 35/101), 25.0% (*n* = 25/100) and 30.3% (*n* = 30/991) at baseline, week 13, 26, 52 and 104, respectively. Of the patients in biochemical remission at week 52, 56.0% (*n* = 14/25) remained in biochemical remission at week 104. In patients with biochemical disease activity at baseline (*n* = 70), biochemical remission was achieved in 28.6% (*n* = 20/70), 34.3% (*n* = 24/70), 21.7% (*n* = 15/69) and 29.0% (*n* = 20/69) at week 13, 26, 52 and 104, respectively.

In patients with clinically active disease at baseline (HBI ≥5), the proportion of patients in biochemical remission was 32.8% (*n* = 22/67), 29.9% (*n* = 20/67), 25.4% (*n* = 17/67), 21.2% (*n* = 14/66) and 27.3% (*n* = 18/66) at baseline, week 13, 26, 52 and 104, respectively. Of patients in biochemical remission at week 52, 64.3% (*n* = 9/14) remained in biochemical remission at week 104. In patients with biochemical and clinical disease activity at baseline (*n* = 45), biochemical remission was achieved in 24.4% (*n* = 11/45), 22.2% (*n* = 10/45), 15.9% (*n* = 7/44) and 27.3% (*n* = 12/44) at week 13, 26, 52 and 104, respectively. Biochemical disease activity levels are displayed in Supplementary Figures 1 and 2, Supplemental digital content 1, http://links.lww.com/EJGH/A821.

#### Endoscopic disease activity

The endoscopic assessment was performed at the discretion of the treating physician. Baseline endoscopic data were available for 49 patients (48.5%), of whom 46 patients showed endoscopic disease activity. In patients that did not show luminal disease activity during endoscopy, three patients started ustekinumab due to a perianal fistula. Thirty-one patients with endoscopically confirmed disease activity at baseline underwent at least one endoscopy during follow-up after a median of 6.7 months (IQR, 4.1–12.6). Two patients showed endoscopic remission after 13.7 months.

In patients with baseline endoscopic disease, corticosteroid-free clinical remission rates were 41.3% (*n* = 19/46), 41.3% (*n* = 19/46), 28.9% (*n* = 13/45) and 31.1% (*n* = 14/45) at week 13, 26, 52 and 104, respectively.

In patients with baseline endoscopic disease and clinically active disease (*n* = 35), corticosteroid-free clinical remission rates were 28.6% (*n* = 10/35), 28.6% (*n* = 10/35), 17.6% (*n* = 6/34) and 23.5% (*n* = 8/34) at week 13, 26, 52 and 104, respectively.

### Ustekinumab dosing

**Fig. 2.**
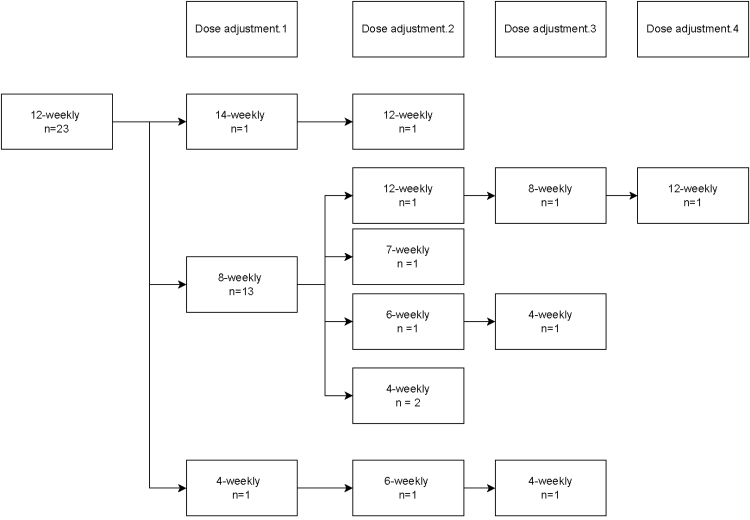
Ustekinumab dose adjustments in all patients initiated on a 12-weekly dosing interval.

**Fig. 3.**
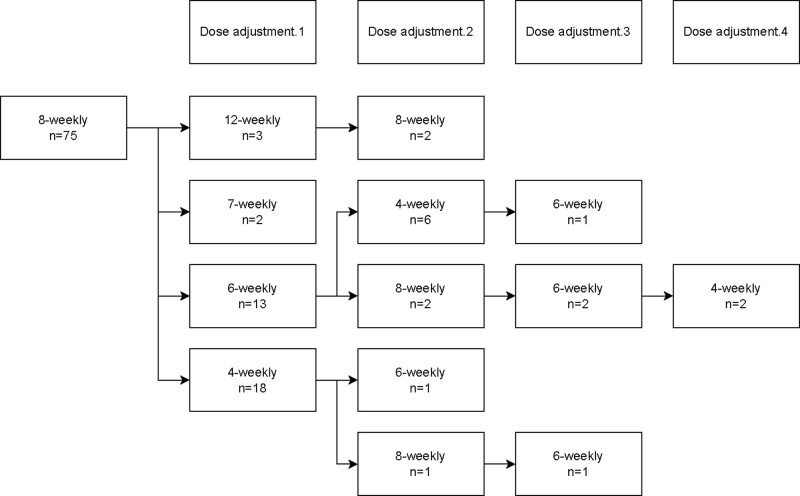
Ustekinumab dose adjustments in all patients initiated on an 8-weekly dosing interval.

After the initial intravenous ustekinumab loading dose, 100 patients (99.0%) were started on ustekinumab maintenance treatment. Of these patients, 75 patients (75.0%) started on an 8-weekly dosing interval, 23 patients (23.0%) on a 12-weekly dosing interval, 1 patient (1.0%) on a 10-weekly dosing interval and 1 patient (1.0%) on 4-weekly dosing interval. Dose adjustments for patients initiated on 12-weekly and 8-weekly dosing intervals are shown in Figs. 2 and 3. Overall, 33 patients (33.0%) required the first dose escalation in the first year of treatment and 15 patients (15.0%) in the second year of treatment. Thirty-nine patients (39.0%) on maintenance treatment required a dosing interval shorter than 8 weeks during follow-up.

In patients initiated on 12-weekly dosing interval, 14/23 patients (61%) initially underwent interval shortening to an 8-weekly interval (*n* = 13) or 4-weekly interval (*n* = 1). The median time to interval shortening was 7.4 months (IQR, 3.0–10.1). The proportion of patients in corticosteroid-free clinical remission was 39.1% (*n* = 9/23), 34.8% (*n* = 8/23), 34.8% (*n* = 8/23) and 22.7% (*n* = 5/22) at week 13, 26, 52 and 104, respectively.

In patients with clinically active disease at baseline (*n* = 16), the proportion of patients in corticosteroid-free clinical remission was 31.3% (*n* = 5/16), 18.8% (*n* = 3/16), 31.3% (*n* = 5/16) and 18.8% (*n* = 3/16) at week 13, 26, 52 and 104, respectively.

In patients initiated on 8-weekly dosing interval, 33/75 patients (44%) initially underwent interval shortening to 7-weekly (*n* = 2), 6-weekly (*n* = 13) or 4-weekly (*n* = 18) interval. The median time to interval shortening was 10.8 months (IQR, 6.2–19.8). The proportion of patients in corticosteroid-free clinical remission was 49.3% (*n* = 37/75), 46.7% (*n* = 35/75), 35.1% (*n* = 26/74) and 40.5% (*n* = 30/74) at week 13, 26, 52 and 104, respectively.

In patients with clinically active disease at baseline (*n* = 48), the proportion of patients in corticosteroid-free clinical remission was 35.4% (*n* = 17/48), 39.6% (*n* = 19/48), 31.9% (*n* = 15/47) and 36.2% (*n* = 17/47) at week 13, 26, 52 and 104 weeks, respectively.

Twelve patients (12.0%) received an additional IV infusion during ustekinumab maintenance treatment due to loss of response after a median of 10.5 months (IQR, 5.3–16.7). At the end of the follow-up, 5/12 patients were still on ustekinumab treatment.

### Safety outcomes

All patients (*n* = 101) were included in the safety analysis and combined for 149 patient-years treatment. During follow-up, 130 ADRs (87.1 per 100 patient-years) were reported. (Table 2) Seven patients (6.9%) discontinued ustekinumab during the first year of treatment due to adverse events: progression of arthralgia (*n* = 2), infusion reaction (*n* = 1), seizure (*n* = 1), frequent infections (*n* = 1), pancreatitis (*n* = 1) and headache (*n* = 1). Infections were the most reported ADR (44.2 per 100 patient-years) for which two patients required hospitalization (1.3 per 100 patient-years). (Table 3) The overall ADR incidence rate was lower in the second year of treatment compared to the first year (43.0 vs. 119.0 per 100 patient-years, respectively).

**Table 2.** Adverse drug reactions related to ustekinumab

	Overall	During first year of treatment	During second year of treatment
Possibly related, *n*	110 (73.7 per 100 patient-year)	84 (97.1 per 100 patient-year)	26 (41.4 per 100 patient-year)
Upper respiratory tract infection	26	19	7
Skin disorder	15	11	4
Flu-like symptoms	12	7	5
Arthralgia	7	7	0
Headache	7	5	2
Infusion-related symptoms	6	6	0
Gastrointestinal disorder	5	4	1
Fungal infection	5	4	1
Other	5	5	0
Ear disorder	4	3	1
Musculoskeletal (other than arthralgia)	4	4	0
Soft tissue	3	2	1
Respiratory tract (other than infection)	3	3	0
Nerve system	3	1	2
Urinary tract infection	3	1	2
Gynaecological	1	1	0
Psychiatric	1	1	0
Probably related, *n*	20 (13.4 per 100 patient-years)	19 (22.0 per 100 patient-years)	1 (1.6 per 100 patient-years)
Infusion-related	10	9	1
Headache	7	7	0
Other	2	2	0
Gastrointestinal	1	1	0
Serious adverse events (possibly/probably related), *n*	6 (4.0 per 100 patient-years)	6 (6.9 per 100 patient-years)	0 (0 per 100 patient-years)
Hypersensitivity/infusion reaction	2	2	0
Herpes zoster	1	1	0
Epileptic seizure	1	1	0
Otitis Media	1	1	0
Pancreatitis	1	1	0

**Table 3.** Infections possibly related to ustekinumab

	Overall	During first year of treatment	During second year of treatment
Mild infection, *n*	43 (29.6 per 100 patient-year)	28 (32.9 per 100 patient-year)	15 (25.1 per 100 patient-year)
Upper respiratory tract infection	16	10	6
Flu-like symptoms	11	6	5
Skin disorder	9	7	2
Ear disorder	3	2	1
Gastrointestinal disorder	3	2	1
Fungal infection	1	1	0
Moderate infection, *n*	19 (12.7 per 100 patient-year)	14 (16.2 per 100 patient-year)	5 (8.0 per 100 patient-year)
Upper respiratory tract infection	10	9	1
Fungal infection	3	2	1
Skin disorder	2	1	1
Urinary tract infection	2	0	2
Flu-like symptoms	1	1	0
Soft tissue disorder	1	1	0
Severe infection, *n*	2 (1.3 per 100 patient-year)	2 (2.3 per 100 patient-year)	0 (0 per 100 patient-year)
Skin disorder	1	1	0
Ear disorder	1	1	0

During ustekinumab treatment 25 patients required at least one hospitalization. In total, 48 hospitalizations were recorded (32.2 per 100 patient-years follow-up). Of note, one patient was hospitalized nine times for repeated perianal surgery (*n* = 6), disease flares (*n* = 2) and intestinal surgery (*n* = 1). In total, eight patients (7.9%) required intestinal surgery.

### Drug survival

**Fig. 4.**
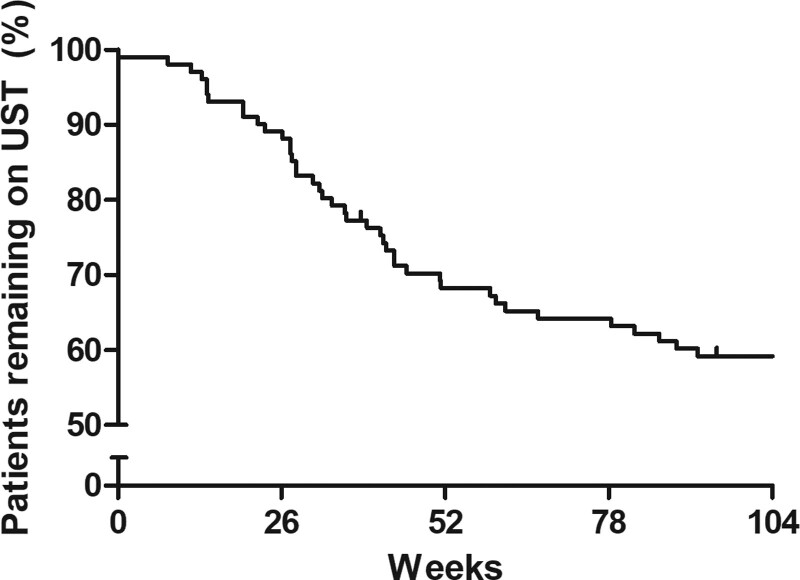
Kaplan–Meier curve of ustekinumab treatment survival during 104 weeks follow-up period. UST, ustekinumab.

Of 101 patients, 41 (40.6%) discontinued ustekinumab after a median treatment duration of 34.0 weeks (IQR, 22.7–51.2). Discontinuation reasons are shown in Supplementary Table 1, Supplemental digital content 1, http://links.lww.com/EJGH/A821. The probability of continuing ustekinumab treatment after 52 weeks was 68.2% and after 104 weeks 59.2%. (Fig. 4) The main reasons for ustekinumab discontinuation were loss of response (*n* = 16), primary nonresponse (*n* = 14) and ADRs (*n* = 7).

## Discussion

In this prospective, multicentre cohort study we evaluated the real-world outcomes of ustekinumab treatment for biological-experienced Crohn’s disease patients during 104 weeks of follow-up. Corticosteroid-free clinical remission rates were achieved and maintained in one-third of all patients and patients with clinically active disease at baseline at week 52 and 104. Of the patients that achieved corticosteroid-free remission at week 52, more than half of the patients maintained this effect throughout week 104. A substantial proportion of patients required dose escalation (48%) which more often occurred in patients initiated on 12-weekly dosing (63%) vs. 8-weekly dosing (48%). Overall, ustekinumab was well-tolerated whereas 7% stopped ustekinumab due to ADR. The drug survival probability was 59% at week 104.

Data on long-term outcomes during ustekinumab use in Crohn’s disease patients is sparse. The long-term extension of the phase III study showed a high clinical response and remission rate [3]. Yet, extrapolation of these outcomes to our study is hampered by methodological differences including strict in- and exclusion criteria. In the last 5 years, three real-world cohort studies have reported long-term clinical and biochemical outcomes beyond 52 weeks. In patients using ustekinumab at week 52 with long-term data available, combined clinical and biochemical remission rates were achieved in 64% at week 72 and 50% at week 88 [20]. These studies were however restricted by a retrospective design and follow-up data beyond 52 weeks was available in only a small number of patients. Recently, a small prospective study showed persistency rates of 77% at week 80 with clinical remission in 88% of these patients and endoscopic improvement in 63% of patients with a follow-up endoscopy [22]. Furthermore, a recent prospective study described outcomes at 104 weeks for 252 Crohn’s disease patients initiated on ustekinumab with a similar design as our study. The authors reported comparable corticosteroid-free clinical remission rates at week 52 and 104 (39 and 34%, respectively), similar to findings in our study (35 and 36%, respectively) [17]. Corticosteroid-free clinical remission rates at week 52 in patients with clinically active disease at baseline are in line with previous findings (24–37% vs. 30% in our cohort) [13,18].

Dose escalation may be attempted to achieve or regain response in case of insufficient primary response or secondary loss of response [30,31]. In our cohort, 48% required dose escalation during follow-up which was most often performed within 52 weeks (overall 33%). No previous study structurally reported dose escalation rates beyond week 52 follow-up. In our cohort, 15% required their first dose escalation beyond 52 weeks. Dose escalation rates during the first 52 weeks of follow-up are in line with those reported in a large Spanish cohort study (28%) [32]. This further underlines that dose optimization is frequently required in this ustekinumab-treated Crohn’s disease population with often refractory disease.

Our study showed that ustekinumab is a well-tolerated long-term treatment for Crohn’s disease. The probability of remaining on ustekinumab after 2 years of treatment was 59%. During follow-up, we observed that the slope of drug survival became more gradual after 52 weeks suggesting that the risk of discontinuation is higher during the first year of treatment compared to the second year of treatment. The main reason for discontinuation beyond 52 weeks was the loss of response (80%). The drug survival rates are comparable with rates reported in previous studies (54–66% at 104 weeks) [11,17,19]. As for drug safety, ADRs probably related to ustekinumab occurred infrequently (13.4 per 100 patient-years) in our cohort. Infections were reported at a rate of 44.2 per 100 patient-year. These safety findings are in line with previous studies [3,17,32]. The overall ADR rate in the second year of treatment was lower than during the first year of treatment, only one probably-related ADR occurred in the second year of treatment and no patient stopped treatment due to ADR in the second year. This suggests that most serious ADRs occur in the early phases of ustekinumab treatment.

Long-term outcomes during ustekinumab use in Crohn’s disease patients are limited. This study provides more insight in the sustained benefit and safety of long-term ustekinumab use. The therapeutic benefit was well maintained beyond 52 weeks up to 104 weeks, while a substantial proportion of patients required dose escalation to achieve or regain therapeutic effect, especially during the first year of treatment. Our data suggest that the risk of ustekinumab discontinuation decreases over time and remains relatively stable in the second year of treatment. These findings are in line with a recent study that showed a lower risk of anti-TNF discontinuation beyond 2-year treatment [33].

The strength of this study was the prospective study design with real-world data of consecutively enrolled Crohn’s disease patients from five Dutch IBD care centres representative for IBD patients in daily practice. However, our study also has some limitations. This study reflects real-world care in which documentation was not always available for all patients. Consequently, we considered missing data as nonresponders and most likely underestimated the true effect of ustekinumab in this study. Furthermore, the HBI score poorly reflects the luminal disease activity in Crohn’s disease patients [34]. In addition, the clinical response may be achieved without inducing clinical remission and vice versa. Objective markers including FCP, CRP and endoscopic assessments are required to provide more accurate information on the disease status [35]. However, in our cohort, only a proportion of patients had an endoscopic assessment (49%) or FCP/CRP (94%) at baseline, and a follow-up endoscopy was performed in 67% and FCP/CRP measurements in 68% at week 52 and 72% at week 104. Future studies on the effectiveness of ustekinumab should include systematic assessments of objective disease parameters such as FCP and endoscopy or cross-sectional imaging.

### Conclusion

This study addressed real-world data on the long-term effectiveness and safety of ustekinumab in Crohn’s disease patients. Our findings illustrate that ustekinumab is a long-term effective and well-tolerated drug for the treatment of Crohn’s disease. More than half of all patients continued ustekinumab treatment 2 years after initiation whereas 36 and 30% achieved and maintained corticosteroid-free clinical remission for all patients and patients with clinically active disease at baseline, respectively. However, a substantial proportion of patients may require dose escalation to achieve and maintain disease remission. Future studies should systematically assess biochemical and endoscopic outcomes.

## Acknowledgements

The authors would like to thank the dedicated IBD nurses and research nurses in each participating hospital.

This study was funded by an unrestricted grant from Janssen-Cilag.

P.T.: conceptualization, collection of data, methodology, statistical analysis and writing; M.v.C.: collection of data, writing, central reading, review final article; R.L.W., M.G.V.M.R., J.M.J.: central reading, review final article; T.E.H.R. and F.H.: conceptualization, methodology, central reading, review final article and supervision).

### Conflicts of interest

RW. has participated in advisory boards, or as a speaker, or consultant for the following companies: Abbvie, Janssen. JJ has served on advisory boards, or as a speaker, or consultant for Abbvie, Amgen, Ferring, Fresenius, Janssen, MSD, Pfizer and Takeda. TR. has served as a speaker, or consultant for Ferring, Janssen and Takeda. FH has served on advisory boards, or as a speaker, or consultant for Abbvie, Celgene, Janssen-Cilag, MSD, Takeda, Celltrion, Teva, Sandoz, and Dr. Falk, and has received unrestricted grants from Dr. Falk, Janssen-Cilag, Abbvie. For the remaining authors, there are no conflicts of interest.

## Supplementary Material
