# Supplementary material for: Long-term effectiveness and safety of ustekinumab in Crohn’s disease: a prospective cohort study
Source: Eur J Gastroenterol Hepatol. 2022 Dec 22;35(3):261–9. doi: 10.1097/MEG.0000000000002506 (PMC9935642; doi:10.1097/MEG.0000000000002506)
Supplement: Supplementary file 1 [file ejgh-35-261-s001.pdf]

Supplementary Files

Effectiveness outcomes for patients previously exposed to vedolizumab

Overall, 32 patients (31.7%) were previously exposed to vedolizumab. Twenty-three patients had clinically active disease at baseline.

Corticosteroid-free clinical remission

In all patients, the proportion of patients in corticosteroid-free clinical remission was 31.3% (n=10/32), 43.8% (n=14/32), 38.7% (n=12/31) and 29.0% (n=9/31) at week 13, 26, 52 and 104, respectively. In patients with clinically active disease at baseline (Harvey Bradshaw Index ≥5), the proportion of patients in corticosteroid-free clinical remission was 26.1% (n=6/23), 30.4% (n=7/23), 36.4% (n=8/22) and 31.8% (n=7/22) at week 13, 26, 52 and 104, respectively.

Biochemical disease activity

In all patients, the proportion of patients in biochemical remission was 31.3 (n=10/32), 34.4% (n=11/32), 31.3% (n=10/32), 12.9% (n=4/31) and 19.4% (n=6/31) at baseline, week 13, 26, 52 and 104, respectively. In patients with clinically active disease at baseline (Harvey Bradshaw Index ≥5), the proportion of patients in biochemical remission was 34.8% (n=8/23), 34.8% (n=8/23), 26.1% (n=6/23), 13.6% (n=3/22) and 22.7% (n=5/23) at baseline, week 13, 26, 52 and 104, respectively.

Drug survival in patients previously exposed to vedolizumab

Of 32 patients, 15 patients (46.9%) discontinued ustekinumab after a median treatment duration of 27.4 weeks (interquartile range [14.1 – 41.7]). Discontinuation reasons included primary non-response (n=5), adverse drug reactions (n=5), loss of response (n=4) and patient preference (n=1). The probability of continuing ustekinumab treatment after 52 weeks was 59.1% and 52.5% after 104 weeks. (Supplementary Figure 1)

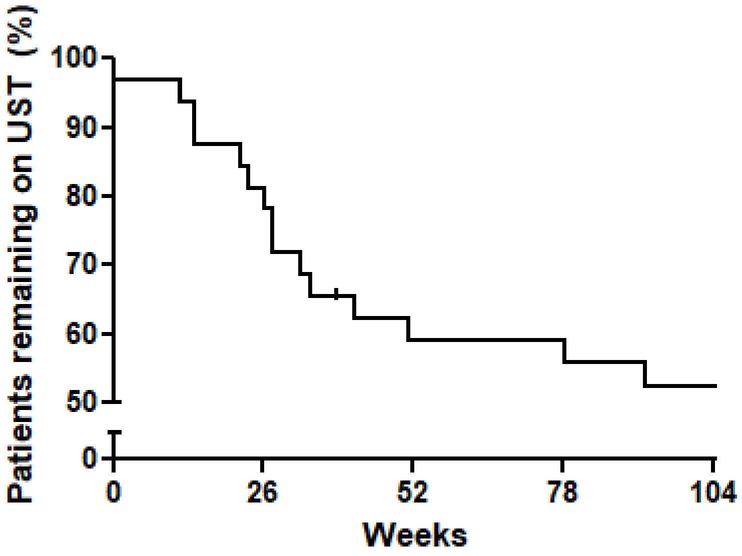

Supplementary Figure 1. Kaplan–Meier curve of ustekinumab treatment survival during 104 weeks follow-up period for patients previously exposed to vedolizumab (n=33). UST = ustekinumab

Reason for discontinuation		Overall<br>N =43~~6~~	During first year of treatment<br>N = 33~~5~~	During second year of treatment<br>N=10~~1~~
**Primary non-response**	N (%)	14 (32.6~~0.4~~)	14 (42.4~~0.0~~)	-
**Secondary loss of response**	N (%)	16~~8~~ (37.2~~9.1~~)	8~~9~~ (24.2~~5.7~~)	8~~9~~ (80.0~~81.8~~)
**Adverse drug reactions**	N (%)	7~~8~~ (16.3~~17.4~~)	~~8~~7 (21.2~~22.9~~)	0 (0.0)
**Request of patient**	N (%)	3 (7.0~~6.5~~)	2 (6.1~~5.7~~)	1 (10.0~~9.1~~)
**Lost to follow-up**	N (%)	2 (4.7~~2.4~~)	1 (3.0~~2.9~~)	1 (10.0~~9.1~~)
**Complications after surgery**	N (%)	1 (2.3~~2~~)	1 (3.0~~2.9~~)	0 (0.0)

Supplementary Table 1. Discontinuation reasons ustekinumab

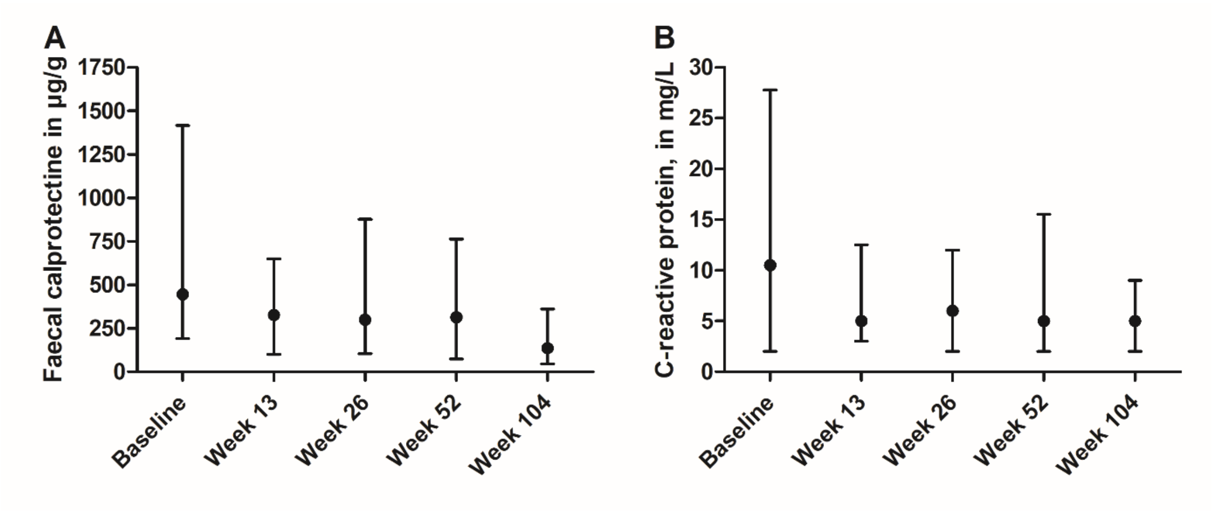

Supplementary Figure 2. Median faecal calprotectin (A) and C-reactive protein (B) levels with interquartile range in the total patient group. Median faecal calprotectin levels of patients on ustekinumab treatment were 445~~8~~ (IQR 190~~195~~ – 1417~~1375~~), 328~~341~~ (IQR 102~~21~~ – 650~~64~~), 300~~340~~ (IQR 105~~120~~ – 877~~877~~), 314~~207~~ (IQR 74~~69~~ – 763~~685~~) and 137~~129~~ (IQR 46~~60~~ – 361~~285~~) at baseline, week 13, 26, 52 and 104, respectively. Median C-reactive protein levels of patients on ustekinumab treatment were 11~~0~~ (IQR 2 – 28~~7~~), 5 (IQR 3 – 13), 6 (IQR 2~~3~~ – 12), 5 (IQR 2~~3~~ – 16) and 5~~4~~ (IQR 2~~3~~ – 9~~8~~) at baseline, week 13, 26, 52 and 104, respectively. IQR = interquartile range.

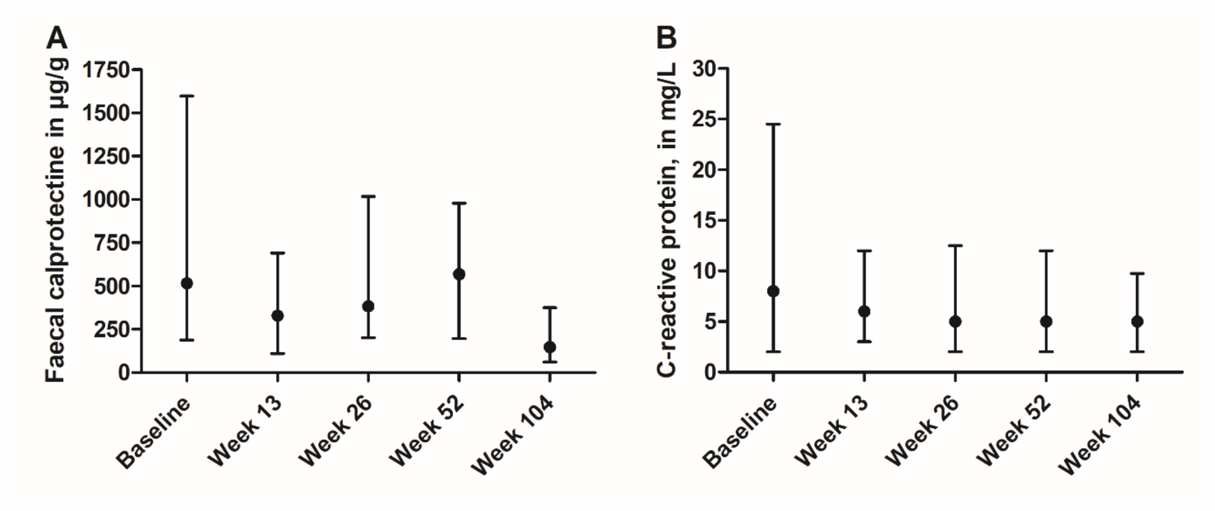

Supplementary Figure 3. Median faecal calprotectin (A) and C-reactive protein (B) levels with interquartile range in patients with clinically active disease at baseline defined as Harvey Bradshaw Index score ≥5. Median faecal calprotectin levels of patients on ustekinumab treatment were 516 (IQR 188 – 1598), 329 (IQR 110 – 690), 383 (IQR 200 – 1018), 569 (IQR 195 – 978) and 147 (IQR 61 – 374) at baseline, week 13, 26, 52 and 104, respectively. Median C-reactive protein levels of patients on ustekinumab treatment were 8 (IQR 2 – 25), 6 (IQR 3 – 12), 5 (IQR 2 – 13), 5 (IQR 2 – 12) and 5 (IQR 2 – 10) at baseline, week 13, 26, 52 and 104, respectively. IQR = interquartile range.
